# Water–Energy–Food Nexus and Life Cycle Thinking: A New Approach to Environmental and Nutritional Assessment of Potato Chips

**DOI:** 10.3390/foods11071018

**Published:** 2022-03-30

**Authors:** Ana Fernández-Ríos, Jara Laso, Francisco José Amo-Setién, Rebeca Abajas-Bustillo, Carmen Ortego-Mate, Pere Fullana-i-Palmer, Alba Bala, Laura Batlle-Bayer, Merce Balcells, Rita Puig, Rubén Aldaco, María Margallo

**Affiliations:** 1 Department of Chemical and Biomolecular Engineering, University of Cantabria, Av. de Los Castros s/n, 39005 Santander, Spain; ana.fdezrios@unican.es (A.F.-R.); jara.laso@unican.es (J.L.); maria.margallo@unican.es (M.M.); 2 Department of Nursing, University of Cantabria, Av. de Los Castros s/n, 39005 Santander, Spain; franciscojose.amo@unican.es (F.J.A.-S.); rebeca.abajas@unican.es (R.A.-B.); carmen.ortego@unican.es (C.O.-M.); 3 UNESCO Chair in Life Cycle and Climate Change ESCI-UPF, Pg. Pujades 1, 08003 Barcelona, Spain; pere.fullana@esci.upf.edu (P.F.-i.-P.); alba.bala@esci.upf.edu (A.B.); laura.batlle@esci.upf.edu (L.B.-B.); 4 Department of Computer Science and Industrial Engineering, University of Lleida (UdL), Pla de la Massa 8, 08700 Igualada, Spain; merce.balcells@udl.cat (M.B.); rita.puig@udl.cat (R.P.)

**Keywords:** carbon footprint, snack, life cycle assessment (LCA), Nutrient-Rich Food 9.3 (NRF9.3), processed product, sustainability, environmental impact, nutritional quality

## Abstract

The water–energy–food (WEF) nexus has become a key concept to promote the cross-sectoral coordination toward sustainable development. In particular, understanding the interdependences of these pillars, as well as addressing a life cycle perspective, is essential when evaluating food production systems. This study explores the environmental impacts and nutritional quality of potato chips, addressing life cycle thinking and a WEF nexus approach. For this purpose, the combined application of life cycle assessment (LCA) and the Nutrient-Rich Food 9.3 (NRF9.3) index was considered to identify the main environmental hotspots and advanced opportunities. The results indicated a major contribution of the cultivation stage on water use, whereas the processing accounted for most of the impacts in energy-related indicators and eutrophication potentials. Improvement opportunities reside in the joint application of drip irrigation, allowing to achieve important water savings, as well as the use of natural gas or pellets instead of diesel, which constitute cleaner energy sources. On the other hand, a poor nutritional density of potato chips became evident from the quantification of the NRF9.3, which can be significantly improved if potatoes undergo a roasted process instead of frying.

## 1. Introduction

**Figure 1:**
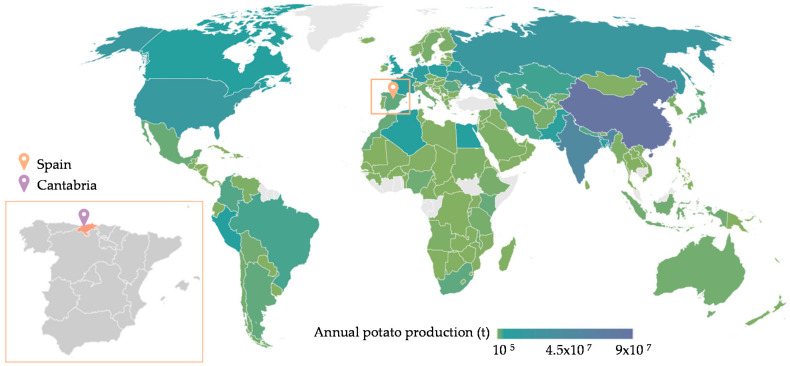
Distribution of potato production worldwide. Data collected from FAOSTAT [2].

Potato (*Solanum tuberosum*) is one of the most popular edible plants in the world [1], ranking third in consumed crops, with an annual global production of 370 million tons [2]. So far, its cultivation has grown unstoppably in developed countries, but it is now expanding strongly in some developing regions [3], driven mainly by its versatility, short maturity period, nutritious characteristics, and employment and income opportunity, making it a resilient cash crop and an indispensable asset against food insecurity [4]. Approximately 80% of the production of this tuber is located in the European and Asian continents (Figure 1), particularly in China (91.8 million tons), India (50.2 million tons), and the Russian Federation (22 million tons), accounting for one-third of the world’s cultivated potatoes [2]. Even though Spain does not have a great contribution at an international level, agricultural activities are essential for the country’s development, constituting the basis for the provision of a set of private and public goods [5]. The harvest of this tuber reached 2.26 million tons 2 years ago, coming up to 273 million and 142 million EUR in exports and imports, respectively [2]. Regionally, Cantabria, a community situated in the north of Spain (Figure 1), presents the highest potato production per cultivated area, getting to 27,600 kg/ha in dry land and 44,000 kg/ha in irrigated area [6], in addition to being the one with the largest per capita consumption, 33.91 kg potato/person and year [7]. From an economic perspective, the main commercial value of this product does not lie in the trade of raw potatoes, but in the added value when they are processed into edible products that appeal to consumers in terms of flavor, texture, appearance, and most of all convenience [8]. In some developed nations, such as Spain, almost one-third of the total volume of potatoes was not consumed fresh in 2019. Of this percentage, around 25% corresponded to processed potatoes, representing an increase of 0.6% in volume and 2% in value compared to the previous year [7].

However, sustainability is not achieved only with economic welfare, but environmental and social aspects are fundamental to find the balance and promote the transition toward a more efficient agri-food sector. Urban activities consume a vast number of resources; the harvesting, processing, and transportation of food require a significant amount of energy, crops, and water [9]. Seeking to understand the interconnectedness of the three sectors of water, energy, and food, so as to improve cross-sectoral coordination in support of sustainable development, the WEF (water–energy–food) nexus has become an important concept [10]. The WEF nexus approach is a holistic vision of sustainability that tries to strike a balance among the different goals, interests, and needs of people and the environment [11]. Understanding and quantifying the WEF interlinkages is particularly important to provide indications on the performance of the system, for which the application of environmental and nutritional tools is essential [12]. 

Food supply chains require systemic and integrated approaches to address the complex dynamics among stakeholders, subsectors, and life cycle stages of products [13]. For this purpose, life cycle assessment (LCA) is recognized as a powerful tool for performing the potential environmental assessment of impacts and resource consumption throughout a product’s life cycle [14]. This tool could provide a consistent analytical framework and environmental data support for industry decision making, identifying best available techniques (BATs) for products [15], and allowing to propose sustainable solutions for global food challenges, as well as supporting the transition toward more sustainable production and consumption patterns [16]. There is no universally recognized methodology for nexus analysis. However, LCA has been extensively applied in analyzing the environmental impact of nexus sectors, aiming to look for effective ways to cope with the current resource shortage and global climate change [17]. Some authors applied this instrument to calculate the carbon footprint of Czech [18] and homemade potato chips [8], as well as the environmental impact by means of several indicators of some processed potato and tomato products [19], or only raw potatoes [20]. On the other hand, a number of studies have been published involving the application of the LCA methodology with a WEF nexus approach, mainly focused on food production, including general food production scenarios [21], or specific products, such as snacks and cereals [22], or cacao [23], as well as energy and fuel production and utilization, such as bioethanol [24] or biogas [25]. On the other hand, in light of the growing concern for food security, it is imperative to introduce tools and indicators considering the nutritional value of food that guarantees personal wellbeing and allows the promotion of healthy dietary patterns. Within this framework, nutrient profile (NP) models have become the basis for regulating nutrition and health claims in the European Union [26]. These indicators enable characterizing food options according to their nutritional quality and have several applications including supporting consumers to make healthy food choices, as well as establishing regulations on the basis of nutrition claims [27], presenting a very useful tool for assessing nutritional aspects considering a nexus approach [28].

To the best of our knowledge, the environmental and nutritional profile under a WEF nexus approach of a wide range of food categories remains unexplored. For this reason, this paper is focused on the application of the LCA methodology and the NRF9.3 (Nutrient-Rich Food 9.3) indicator to explore the issues of the WEF nexus of a well-known and increasingly consumed processed food, namely, potato chips. For this purpose, an estimation of the water and energy consumption, carbon footprint, and nutritional quality of this product was carried out. The novelty of this study lies in the fact that, unlike previous works that analyzed the environmental performance of this product by means of a single [8,18] or several indicators [19,20], it takes a broader approach by means of the WEF nexus, introducing the nutritional variable as a crucial aspect in food evaluations. The joint assessment of the WEF aspects will allow understanding the complex interlinkages between these pillars and determining the main hotspots. In addition, on the basis of these critical points, some recommendations are suggested, and an analysis of alternative scenarios is carried out, in which improvements focused not only on the cultivation but also on the processing stage are proposed, evidencing that a modification in the production process can lead to a change in both the environmental profile and the quality of the food, highlighting the key role of the nexus application.

## 2. Materials and Methods

The methodology followed in this study was based on life cycle thinking; therefore, the guidelines established in the UNE EN-ISO 14040 standard [29], related to the principles and framework of LCA, were considered. This procedure is divided into four steps: (i) goal and scope definition, (ii) life cycle inventory, (iii) life cycle impact assessment, and (iv) interpretation. In this study, a combination of the life cycle environmental impacts in terms of energy and water was developed by applying LCA, as well as the estimation of the nutritional quality of the product using the NRF9.3 model.

### 2.1. Goal and Scope

The methodological framework involves the definition of the functional unit (FU), function of the system, system boundaries, assumptions and limitations, and allocations, among other characteristics. Although the overall objective of the work included the evaluation of both environmental and nutritional aspects, the goal of the LCA was to calculate the water and energy impacts associated with potato chip production, constituting the water–energy issues of the WEF nexus. The novelty of this study is justified by the fact that it is the first application of the WEF nexus perspective to this food product, providing a basis to suggest possible advance actions in the sector and establishing guidelines for further analysis.

The overall function of the system is the production of potato chips; hence, the FUs should reflect this purpose. The most common FUs used in the environmental analysis of food are weight-based FU (WBFU), energy-based FU (EBFU), and nutrient-based FU (NBFU) [30]. The former, which usually provides the impact per 1 kg of food, especially for agricultural products [31], allows analyzing only the environmental burdens of the product or process, facilitating the comparison with other reference values, while the last two consider nutritional aspects, normally expressed by kcal (EBFU) or per g of protein (NBFU). In this case, taking into account that one subobjective of the study was to find the environmental hotspots of the production process, a WBFU was defined. Moreover, given that the object under study is a product marketed by a company, the FU was selected according to the format of the most-selling bag, containing 50 g of potato chips, and its corresponding primary packaging (polypropylene bag). This FU is also considered appropriate as it would facilitate the process of supplying information to consumers through eco-labeling, since it provides the value of the impact associated with the bag of potatoes they are consuming, and its interpretation would be simpler than in the case of an FU of 1 kg of potato chips.

**Figure 2:**
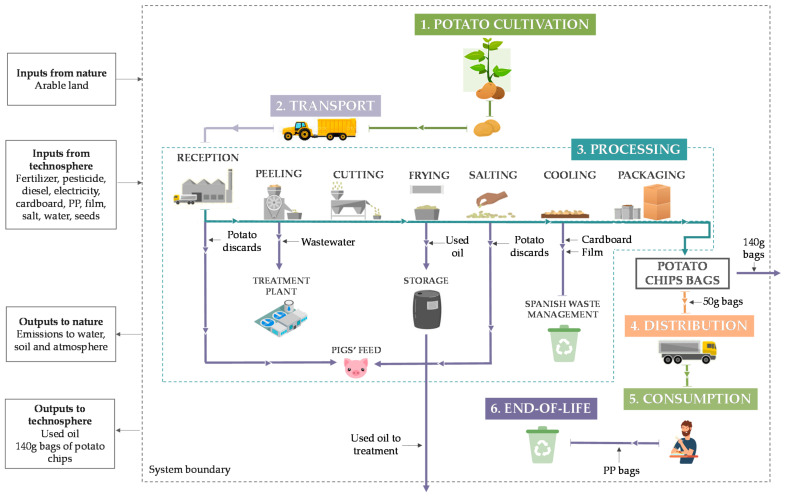
Flow diagram of the system under study.

The system boundaries represent the interface between the product system and the environment, and their definition determines which unit processes shall be included within the assessment. The limits included all stages of the life cycle of the product, i.e., from the raw material extraction (potato cultivation) to the end of life (EoL) of the packaging, considering what is known as a ‘cradle-to-grave’ approach (Figure 2). Pollutant emissions generated in the production system and management of intermediate waste were included in the assessment. On the contrary, the construction of the infrastructure, as well as its maintenance, was excluded from the study due to the low influence of this stage on the total impact.

Briefly, the product life cycle can be described as follows: the first stage involves the cultivation of the crop, requiring protective chemicals (fertilizers and pesticides) to guarantee the quality of the product, as well as water for the sprinkler irrigation and fuel for agricultural machinery used for fertilization and harvesting. Afterward, the transport of potatoes to the factory, located at 3 km, is carried out in a tractor. Within the production process itself, the first step consists of the reception and peeling of the potatoes, which is performed in a peeler with rough and sandpaper walls. The resulting water with peels is discharged directly down the drain and later managed in a wastewater treatment plant. In addition, at this stage, a selection of the raw potatoes is carried out, discarding those that are in poor condition as feed for pigs. Subsequently, cutting is accomplished by means of a centrifugal bowl that laminates the potatoes and sends them to a fryer with sunflower oil, which is heated by a diesel generator. Once the oil does not have the sufficient quality to continue being used, it is deposited in drums that are later collected by a manager. The fried potatoes are salted and transported by a belt in which the smallest peaks are discarded. After they have cooled for 24 h in cardboard boxes to remove excess oil, potato chips are packaged in 50 g or 140 g bags. A peculiarity of these bags is that they are composed of only one material, polypropylene, as opposed to others commonly used that utilize both an external plastic and an internal aluminum layer. This fact makes their EoL simpler by not having to separate different materials for their management, presenting a good eco-design practice that provides benefits to both the company and the environment. These potato chips bags are placed in cardboard boxes and distributed in a van around the region, within a maximum radius of 100 km. This regional sale is also an important environmental advantage, as it avoids long distances and relies on local products. Finally, the waste management of the plastic bags thrown away after consumption is carried out on the basis of the Spanish treatment mix, as well as that of the cardboard, involving the following technologies: 1% open burning, 32% unsanitary landfill, 48% sanitary landfill, and 19% municipal incineration [32].

The scope of the LCA also includes the definition of allocation procedures. Allocation of flows, emissions, and waste is crucial in industrial or production processes in which co-products or intermediate wastes are generated. In this case, two different allocation hotspots were identified in the system under study. On the one hand, allocation is necessary in the cultivation stage due to the generation of unwanted products or residues, i.e., discarding bad potatoes. Mass-based allocation was considered the most appropriate approach since both the desired and the undesired products are obtained from the same process (same crop). In addition, other allocation strategies, e.g., economic or consumption-based, were discarded since waste has no economic benefit and is not intended for human consumption. Hence, it was assumed that 98.81% of the environmental impact was assigned to the raw potatoes destined for chip production, whereas the remaining 1.19% was allocated to the discards. On the other hand, an assignation was defined in the processing stage due to the production of coproducts (140 g bags) and generation of residue (potato chip discards). In this case, a mass allocation was also considered, as it seems to be the most adequate taking into account the waste generation that, unlike the coproduct, does not have economic value in the market. Therefore, the allocation procedure led to an assignation of 32.66% of the environmental burden for potato chips contained in 50 g bags, 65.33% for chips in 140 g bags, and the remainder (2%) for potato chip discards.

### 2.2. Data Collection, Hypothesis, and Life Cycle Inventory

The life cycle inventory (LCI) involves the compilation and quantification of inputs and outputs of the system throughout its life cycle. On the one hand, primary information, i.e., foreground data, were obtained from the producer factory located in the south of Cantabria, representative of this region. The industry under study reported data by means of a questionnaire, comprising information of the capital goods, as well as operational data for the cultivation, processing, and transportation stages of the year 2019. On the other hand, secondary data, i.e., background processes related to energy or raw materials, were compiled from the Ecoinvent v3.7.1 and Agribalyse v3.0.1 databases.

Given that LCI is the trickiest stage of the LCA methodology, since the LCA practitioner often must face problems with missing inventory data [33], a set of assumptions were made in order to get an inventory as detailed as possible. Although these hypotheses may be a source of uncertainty in the LCI, they are necessary to fill gaps in the data provided by the producer. The company reported that a nitrogen-based fertilizer is used. Hence, potassium nitrate was assumed since it is one of the most used in agriculture, particularly for potato cultivation, which generally requires an external supply of potassium, as well as nitrogen, to obtain high yields and good quality [34]. On the other hand, information regarding the pesticide was unknown for the company since the cultivation of potatoes is carried out by an external producer; thus, an unspecified pesticide from the Ecoinvent database was considered. This decision was made on the basis of the small amount of insecticide required (maximum 2.5 kg per year) and its estimated low contribution to the total impact. Table 1 presents the LCI for the production of potato chips, considering an FU of a 50 g bag of potato chips. Equivalences between materials reported by the producer and processes of the Agribalyse and Ecoinvent databases are reported in Table A1, contained in Appendix A.

### 2.3. Environmental Impact Calculation

**Figure 3:**
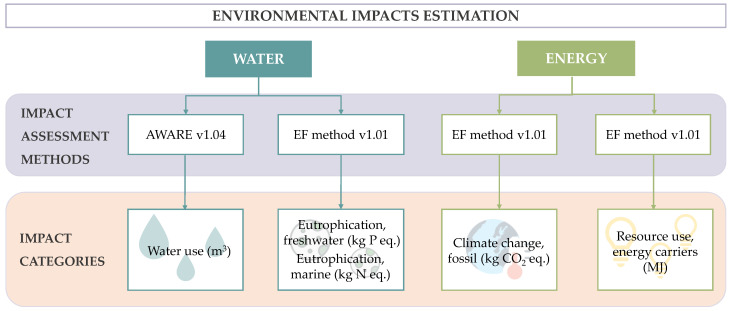
Impact assessment methods and indicators used for the calculation of environmental footprints.

This section corresponds to the life cycle impact assessment (LCIA). The software used to compute this phase was SimaPro v8.3. In order to analyze the WEF issues, different impact methods and indicators were applied, as Figure 3 illustrates. The water footprint (WF) is a methodology to determine the impact on water resources by individuals, communities, businesses, as well as production processes [35], i.e., the amount of freshwater used to develop an activity or to produce a good. The available water remaining (AWARE) method was applied to be used as water use midpoint indicator, representing the available water remaining per area in a watershed after the demand of humans and aquatic ecosystems has been met [36], assessing the potential of water deprivation [37]. In addition, in this study, other two indicators quantifying the degradation of aquatic ecosystems, namely, freshwater eutrophication potential (EPf) and marine eutrophication potential (EPm), were analyzed. EP (eutrophication potential) is defined by the eutrophication process, which begins with increases in nutrient loading to water ecosystems, typically limited by nitrogen or phosphorus [38]. For this purpose, the environmental footprint method was applied since it provides a higher degree of specificity and consistency than other methods, establishing minimum data quality requirements [39]. This method was also applied to determine energy-related issues. The indicator ‘resource use, energy carriers’ (RU), expressed in MJ, was considered to quantify the use of abiotic resources, in this case, fossil fuels, as an approximation of the energy consumption of the system [40]. Lastly, the ‘climate change, fossil’ (CC) indicator was used to determine the GHG emissions from fossil resources emitted to the atmosphere during the life cycle of the product, assessing the radioactive forcing as global warming potential for a 100 year period. Although this impact category does not evaluate energy consumption, it was considered an interesting variable to be included in the energy footprint since it is directly related to the use of fossil resources.

### 2.4. Nutritional Quality Estimation

The NRF9.3 model, developed by Drewnowski [26], was used to assess the nutritional quality of the product, as it seems to be the most appropriate indicator within a WEF nexus approach [28]. The NRF index is a formal scoring system that ranks foods on the basis of their nutrient content, helping consumers to identify nutritious foods and design healthier diets [41]. It is based on two subscores: NR9 (nutrient-rich 9), which evaluates nine nutrients to encourage (protein, fiber, vitamins A, C, and E, calcium, iron, potassium, and magnesium), and LIM (limited nutrient score) that considers three nutrients that should be limited (sodium, saturated fats, and added sugar), as described in Equation (1).

(1) $$\;NRF9.3_{kcal}=(\underset{1-9}{\sum }\frac{nutrient_{i}}{DV_{i}}\cdot 100)/ED-(\underset{1-3}{\sum }\frac{L_{i}}{MRV_{i}}\;\cdot 100)/ED$$

where *nutrient_i_* is the content of nutrient *i* in 100 g of edible portion, *DV_i_* is the daily value for nutrient *i*, *ED* is the energy density, *L_i_* is the content of limiting nutrient *i* in 100 g of edible portion of food, and *MRV_i_* is the maximum recommended value for nutrient *i*. For this study, the NRF9.3 under an energy basis was selected as it best reflects the concept of nutrient density.

The nutrient content of potato chips was sourced from the BEDCA database (Spanish Food Composition Database) [42], whereas the DVs and MRVs, reported in Table 2, were compiled from the European Commission (EC) Regulation no 1169/2011, related to food information provided to consumers [43] and the report ‘dietary reference values for nutrients’, published by EFSA (European Food Safety Authority) [44]. The values considered were based on the recommended intake for an average adult, assuming a daily diet of 2000 kcal.

## 3. Results

### WEF Nexus in Potato Chip Production

**Figure 4:**
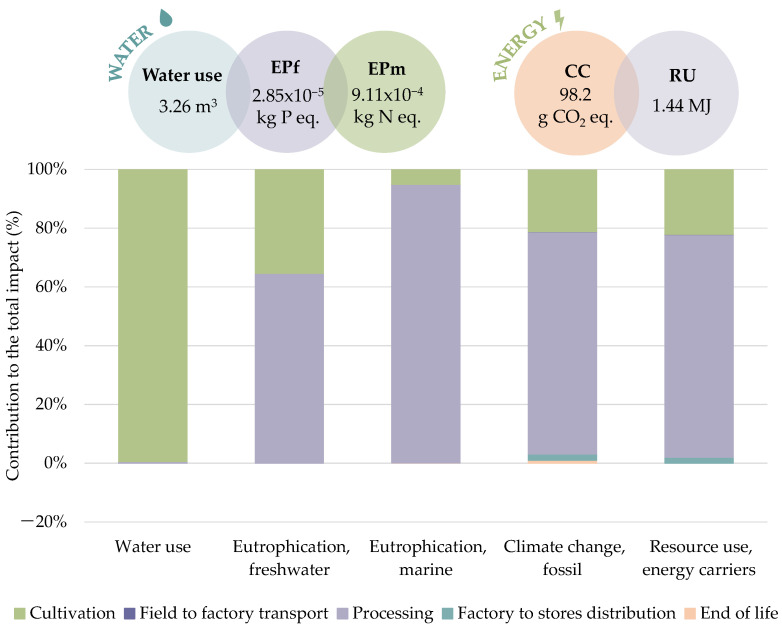
Water- and energy-related indicators of potato chips production. At the top, the total value of each indicator is provided per FU; at the bottom, the contribution of each life cycle stage to the environmental impact categories is presented.

Figure 4 illustrates the water and energy-related indicators, encompassing both total consumptions and relative contributions of each life cycle stage. The water scarcity footprint, i.e., water consumption in potato chip production, was estimated at 3.26 m^3^ of water deprived per FU (50 g bag). The cultivation stage was recognized as the main carrier of water stress, with a relative contribution of 99.69%, mainly coming from crop irrigation requirements. A contribution lower than 1% was found for the processing, involving both transformation and packaging, chiefly caused by the sunflower oil, cardboard, and polypropylene production. It is worth noting the regionalization influence of the AWARE method, which takes into account the water availability and demand of a specific geographic location, making the impacts highly dependent on these factors. In this case, results were provided considering Spanish tap water, as well as national waste management strategies. Comparing the water use estimated with the deprivation potential assuming European resources, a consumption of approximately twice was obtained at a national level (3.26 m^3^ versus 1.72 m^3^). This is explained by the fact that, although there are higher scarcity problems than the world average in both areas, the shortage is much more significant in Spain, leading to major consumption in this region. On the other hand, an EPf of 2.85 × 10^−5^ kg P equivalent/FU was achieved, with cultivation (35.44%) and processing (64.61%) stages identified as the major contributors. Tap water for irrigation, cardboard, diesel, and sunflower oil was recognized as the most critical resource. Similarly, EPm accounted for 9.11 × 10^−4^ kg N equivalent/FU, with the processing and cultivation being responsible for 94.76% and 5.08% of the total impact, respectively. In this case, the production and application of fertilizers and pesticides represented almost half of the cultivation burden, whereas the other half was attributed to irrigation water and potato seeds. In both EP indicators, transport presented negligible impacts (lower than 1%), as well as in the EoL of packaging, whose contribution was totally insignificant.

The average CC of potato chips was calculated as 98.2 g CO_2_ equivalent per bag, with transformation (75.63%) and cultivation (20.98%) identified as hotspots. The production of sunflower oil and the diesel burned in the generating set were recognized as critical resources, representing 55.9% and 25% of the GHG emissions of processing, respectively. Tap water and fertilizer accounted for more than 50% of the cultivation burdens. The same trend was observed for the category measuring fossil resource use, which reached 1.44 MJ/FU. Both indicators had contributions to the transportation stages ranging from 0.09% to 2.08%, indicating that transport, at least of raw materials, could be considered negligible in further analysis. Furthermore, the packaging EoL had a contribution up to 1.1%.

Similar results were reported by Parajuli et al. [19], who assessed a cradle-to-grave environmental analysis of potato and tomato processed products, and Frankowska et al. [20], who analyzed the impacts of vegetables consumption. Both papers identified potato cultivation as the most critical stage in water consumption, whereas the processing led the EPf. Likewise, Ridoutt et al. [45] published that 96% of the water availability footprint was produced by irrigation. Regarding the energy consumption, important impacts occurred at the storage stage, due to the refrigeration of raw potatoes [45], which was not considered in our study since they are stored at room temperature. However, the processing stage also turned out to have important contributions in RU and CC in these studies. Comparing the total values, the carbon footprint estimated in this work (equivalent to 1.96 kg CO_2_ eq./kg potato chips) was similar to that reported by Ponsioen and Blonk [46] of 2.1 kg CO_2_ eq./kg, and Moudrý et al. [18] of 2.07 kg CO_2_ eq./kg, while it was somewhat different to the 3.8 kg CO_2_ eq./kg and 0.85 kg CO_2_ eq./kg, calculated by Frankowska et al. [20] and Parajuli et al. [19], respectively.

**Figure 5:**
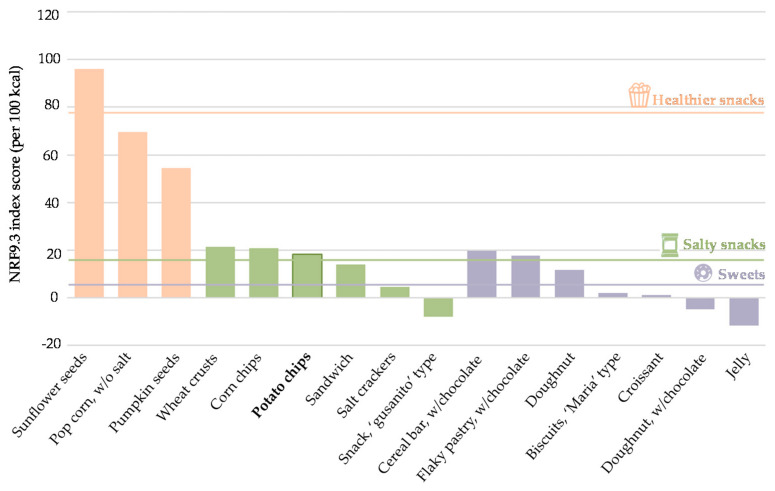
NRF9.3 index scores for different snack options. Bars represent the NRF9.3 for each food, while horizontal lines show the average NRF9.3 for healthy, salty and sweet snacks.

Regarding the nutritional aspects, the NRF9.3_100kcal_ score of potato chips reached 18.39, quite similar to the 23 reported by Hess and Slavin [47] and 19.3 calculated by Hess et al. [48], who, in their article, applied the NRF10.3 model. In order to have a wider perspective and give meaning to this value, this index was calculated for different snacks, including five additional salty options, seven sweet snacks, and three healthier alternatives (Figure 5). The NRF9.3 scores varied between −11.74 (jelly) and 95.96 (sunflower seeds). For salty snacks, which includes the product under study, the overall average NRF9.3 score was 11.86. Results for this food category ranged from 21.27 (wheat crusts) to −7.99 (corn appetizer, ‘gusanito’ type), which received a negative value, indicating a larger amount of nutrients to limit than nutrients to encourage. In light of these points, potato chips are situated in a relatively high position within this rate of scores, showing that they are not an unfavorable option. However, compared with other food categories, such as fruits, with NRF9.3 scores between 44 [47] and 93.78 [49], vegetables (average 129) [47], or beans (46.73) [49], the low nutritional density of this processed product becomes evident. Despite this, sweet snacks appear to be even less healthy than salty ones, accounting for an average score of 5.12. This category is undoubtedly the one that obtains the lowest score, since, regardless of the positive nutrients, it is severely penalized by the high added sugar content, indicating the most nutrient-poor foods. Two sweet options were situated slightly above 0, whereas another two were significantly below, evidencing their low nutritional density. The only exception that ranked slightly above potato chips was represented by cereal bars with chocolate, with an estimated score of 19.65, a value that may be surprising given that they are commonly known among consumers as a healthy snacking option. Accordingly, sunflower or pumpkin seeds and popcorn without salt and oil were identified as the healthiest snacks, mainly due to the lack of added sugar, relatively low content of salt and saturated fats, and important amounts of protein, vitamins, and minerals. In relation to popcorn, it is worth noting the influence of the lack of oil or salt content; otherwise, its NRF9.3 score drops drastically to 8 [47], making it a worse choice than potato chips.

## 4. Opportunities for Improvement and Recommendations

Some of the pressure on potato chip production may be alleviated by the sustainable use of natural resources, as well as the application of innovative technologies to reduce environmental impacts. Given that the first step in mitigation is estimating impacts, LCA allowed identifying critical stages and materials, enabling the proposal of sustainable solutions, and supporting the transition toward healthier and more environmentally friendly production and consumption patterns. According to the results of the study, the environmental hotspots that should be targeted for improvements were focused on the processing and cultivation stages. Section 4.1, Section 4.2 and Section 4.3 present three potential alternatives that could bring environmental and nutritional benefits to the product under study.

### 4.1. Implementation of Drip Irrigation

Surface irrigation turned out to be the major contributor to water footprint, as potato is a very sensitive crop to water stress. To optimize yields, the available soil water should not be depleted by more than 30–50%, and the soil should be maintained at a relatively high moisture content [50]. Although some authors reported that a solution could reside in a slight reduction in water supply, as around 20% of water can be saved without affecting potato yield [51], there are other methods to practice water saving in agriculture that achieve higher water use efficiency, increase crop yields, and improve the quality of the tuber [52]. In this context, the application of drip irrigation presents a great opportunity to decrease the water footprint of the system, always guaranteeing the necessary water supply. Drip irrigation is the recommended irrigation strategy in Mediterranean areas for potato production [52], and it could reduce the amount of water by 30% to 60% (compared to conventional irrigation), in addition to decreasing soil moisture evaporation and preventing weed growth by supplying water mainly to the root zone [53].

**Figure 6:**
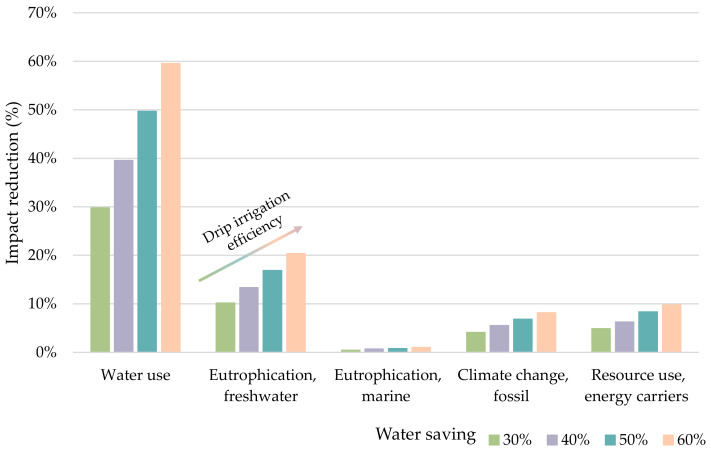
Reductions in water and energy-related indicators considering different drip irrigation scenarios.

The environmental impacts of the production system were recalculated applying different scenarios based on percentages from 30% to 60% of water saving. The WF of the system in an optimistic scenario (60% of water saving) reached 1.32 m^3^, achieving a reduction of 59.51% with respect to the baseline scenario (sprinkler irrigation) (Figure 6). EPf and EPm accounted for 2.27 × 10^−5^ kg P and 9.02 × 10^−4^ kg N eq. per FU, leading to reductions of 20.35% and 0.99%, respectively. Energy-related indicators achieved impact reductions of 8.15% (CC) and 9.72% (RU). Taking into account 30% of water saving in irrigation (pessimistic scenario), water use of 2.29 m^3^ per FU was estimated, reducing the total water footprint by practically 30% (29.75%), as this source was identified as the main contributor to water stress. EPs of 2.56 × 10^−5^ kg P eq. and 9.07 × 10^−4^ kg N eq./FU were calculated for freshwater and marine water, respectively, resulting in 10.18% and 0.44% total reductions. It is worth noting that the impact of EPm barely decreased in both scenarios, which is due to processing having a much greater contribution to this impact category than cultivation; thus, it is mandatory to focus efforts on finding sustainable solutions applicable to this stage. Lastly, CC achieved a reduction of 4.07% (94.2 g CO_2_ equivalent/FU), whereas that of RU was 4.86% (1.37 MJ/FU). Likewise, for energy-related indicators, the implementation of drip irrigation did not lead to significant improvements since the processing stage was the main cause of environmental degradation. In view of these results, it is evident that a higher efficiency of drip irrigation would avoid larger burdens. However, this depends on different factors, such as soil properties, salinity levels of irrigation water, or the climate of the area of cultivation [53]; hence, the expected yield cannot be known with certainty with the data available in this study. This improvement opportunity includes engaging and influencing farmers to improve agricultural practices to reduce the environmental impacts of their agriculture activities. However, this action supposes an important economic investment, making essential the support of policy managers, through actions and measures, such as agri-environmental programs (AEP), which support environmentally friendly agricultural production systems and help to preserve endangered species, habitats, plants, and animal genetic resources, as well as increase the share of buffer land in the agriculture landscape [54].

### 4.2. Application of Natural Gas and Pellets as Heat Source in Processing

In line with the above, raw potato processing turned out to be the largest consumer of fossil resources, as well as the main source of emissions and eutrophication. The use of diesel was identified as one of the major hotspots of the transformation, leading to important environmental degradation in terms of water and energy. For this reason, two alternative resources to heat the vegetable oil were considered in an alternative analysis with the aim of evaluating their influence on the sustainability of the overall system. On the one hand, the use of natural gas for power generation is quite significant today, providing a clean technique for the energy supply [55]. Given that this resource presents the best environmental performance among fossil fuels [56], it was selected for the assessment. On the other hand, biomass is considered an appropriate source of energy to gradually substitute fossil fuels, which could play an important role in the reduction in GHG concentrations [57]. Pellets are characterized by higher energy density and lower moisture content than other raw biomass, leading to higher effective heating values; thus, their application stands out in terms of heat and energy generation [58]. For these reasons, the substitution of diesel for pellets was also taken into account.

**Figure 7:**
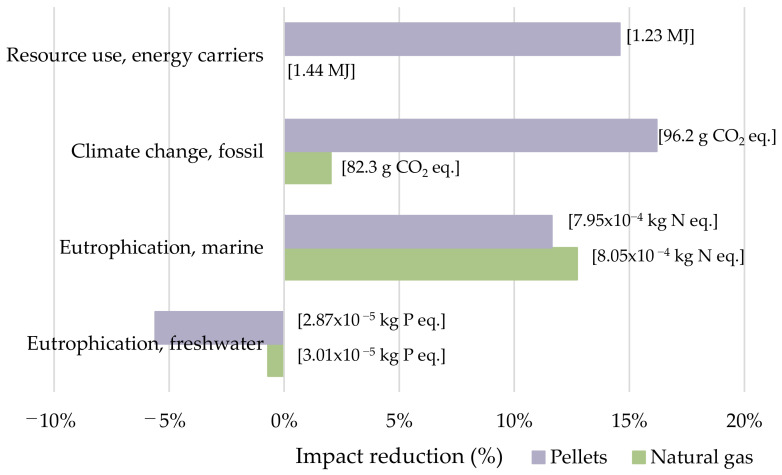
Reductions in water and energy-related indicators considering alternative energy resources for frying. Values between brackets represent total impacts per FU.

Figure 7 depicts the environmental impact reductions in terms of RU, CC, and EP of the two alternative energy resources for the frying. Water use was not included in this assessment because more than 99% of the impact came from cultivation. Pellets presented important environmental benefits in almost all impact categories; RU was reduced by 14.58%, CC was reduced by 16.19%, and EPm was reduced by 11.64%. On the contrary EPf increased by almost 6%, a percentage that can be afforded taking into account the important reductions in other categories. For its part, natural gas managed to reduce the impacts in CC (2.04%) and EPm (12.73%), whereas RU remained at the same value. Even though the use of fossil resources is lower for the production of energy from natural gas than for diesel, the contribution to the total impact is not large enough to report reductions.

Although a significant investment is required for the replacement of these technologies at an industrial level, they will lead to a substantial improvement in the sustainability of the production process, so that the replacement of diesel by one of these two alternatives is highly recommended. On its own, this opportunity would bring significant environmental benefits to the industry under study. However, combined with the application of drip irrigation, very significant reductions in the burdens of all indicators can be achieved. Hence, it is strongly recommended that these measures be further explored, including technoeconomic feasibility in order to evaluate the potential of the actions as a whole.

### 4.3. Replacement of the Conventional Oil Fryer with a Hot-Air Fryer

At the environmental level, but also influencing the nutritional quality of the product, a potential improvement opportunity includes the substitution of the stovetop deep fryer by a hot-air fryer. Hot-air frying cooks potatoes through the circulation of superheated air, reducing the high fat content of the final product, while increasing the content of other nutrients [59]. To simulate this alternative, the electricity consumption for the fryer was estimated according to Carvalho et al. [8], who assessed the GHG emissions of two frying processes for potato chips. Accordingly, the consumption of sunflower oil, diesel, and cardboard boxes used to eliminate the excess oil was removed from the modeling.

**Figure 8:**
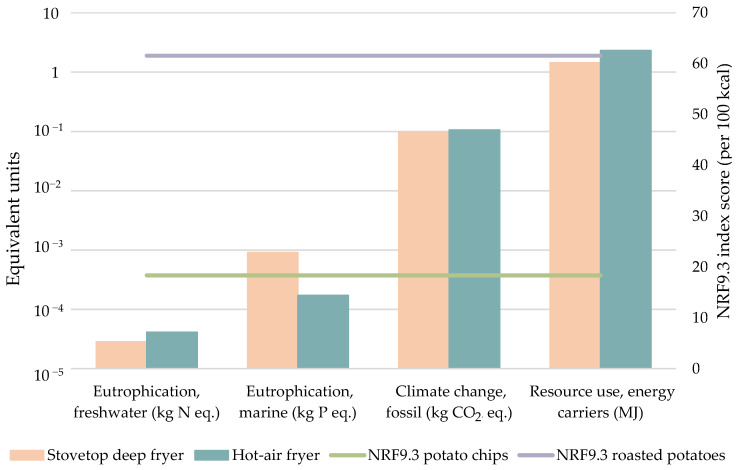
Comparison of two different frying techniques in terms of water, energy, and food indicators. Bars represent the environmental impact, while horizontal lines show the NRF9.3 score of each final product.

The use of this innovative technology led to a varied trend of results (Figure 8). Water use was not assessed as it was hardly influenced by the transformation. While EPm reported higher burdens by the conventional frying technology due to the use of cardboard, sunflower oil, and diesel, EPf reported a higher burden for the hot-air frying due to the great amount of electricity consumed in this process. Likewise, energy-related indicators experimented a growth for the hot-air fryer compared to the stovetop deep fryer associated with the energy consumption. CC reached 106 g CO_2_ eq./FU, translated into a 7.9% increase, whereas for RU the rise was more significant, by around 61%. In light of the values, the use of this technology is not appropriate from an environmental perspective, leading to a worse performance of the system. However, from a nutritional point of view, the results were opposite. The NRF9.3_100kcal_ score achieved for roasted potatoes, 61.57, evidenced that this product presented superior nutritional qualities than potato chips, which accounted for a value of 18.38. Potato products have a wide range of nutrient-densities depending on the type of preparation and cooking techniques, which usually cause the loss of original nutrients of the raw tuber, which has an NRF9.3 score of 83.89. When subjected to frying, some natural nutrients are lost, whereas, at the same time, the additional ingredients added to potato, i.e., sunflower oil, provide it with extra positive nutrients such as vitamins or minerals. On the contrary, roasted potatoes do not contain additional ingredients, as they are cooked only with superheated air; hence, the modification of nutrients is only associated with processing. Despite this fact, the main difference in the NRF9.3 scores of both products lies in the nutrients that should be limited; roasted potatoes contain 0.026 g of saturated fatty acids and 21 mg of sodium, while those quantities in potato chips sum to 7.7 g and 700 mg, respectively, thus penalizing the latter. In addition, frying produces a significant enlargement of the total energy of potatoes (538 kcal/100 g), leading to inferior scores compared to low-energy-dense foods, such as roasted potatoes (206 kcal/100 g).

At this point, it is mandatory to highlight the influence of energy density in the estimation of NRF9.3 scores. Considering that a weight or an energy basis can lead to significant changes in results, a system based on 100 g makes no allowances for the fact that different foods and beverages are consumed in very different amounts, giving overly favorable scores to sugared products [26]. On the other hand, a 100 kcal basis tends to overrepresent energy-dense foods, giving overly high scores to very-low-energy-dense foods [60]. In this study, since a snack was assessed, which is not usually consumed in large amounts and whose energy supply could represent a significant percentage of the recommended daily energy intake, a 100 kcal basis was considered in order to better reflect the concept of the nutritional density of foods. However, depending on the type of food or, especially, diets as a whole, it would be strongly recommended to study different calculation bases to lead to the most suitable and representative results.

In summary, this improvement opportunity includes consumer involvement, and only raising the awareness of the implications of their choices could help to improve their quality of life and promote their health. The first step must be consumer acceptance to change dietary habits and consumption patterns, switching to healthier food products. Although sometimes the most health-conscious consumers tend to buy and eat potato chips that claim to be light, this snack turns to be even more nutritionally poor than conventional potato chips, accounting for an NRF9.3 score of 15.80. Although it has lower caloric content than conventional potato chips (100 kcal less per 100 g) and a slight reduction in saturated fats, the sodium content is significantly higher, penalizing this product. In addition, it is important not to forget the preference to modify these habits toward more sustainable and environmentally friendly food products. The application of eco-labeling to provide relevant information about the production and the ecological impact of a product plays an important role in promoting sustainable consumption patterns [61], as well as influences and incentivizes the producer to develop greener products that consumers value differently than conventional ones [62]. At this point, incorporating consumers’ preferences into discussions and sorting out the difficult tradeoffs that affect actual health and environmental outcomes in a consistent way with consumer preferences represent important challenges for the future [63].

## 5. Limitations and Perspectives

The WEF nexus perspective helped address water, energy, and food resource challenges, applying detailed analysis under a life cycle thinking that enabled the improved provision of multi-sector nexus solutions through decision making, maximization of resource-use synergies, and sustainable outcomes achieved through socially and technically feasible strategies [64]. On the one hand, the application of LCA provided a comprehensive assessment tool, highlighting potential environmental tradeoffs, taking the first steps toward future research, and advancing the knowledge base. On the other hand, the use of NP models, particularly the NRF9.3, presented a novelty within the WEF nexus approach, highlighting not only food security but also nutritional aspects.

Among the limitations of the present work, one of the main problems faced was the uncertainty associated with the quality of the inventory data. Although LCI compilation is considered a rather straightforward procedure, the unavailability of inventory information is a common hurdle encountered in LCA, which is often overcome by making use of secondary data [65]. In this case, assumptions had to be made on the basis of the current literature, which provides information that is closer to reality, but is still not fully certain. However, it is evident that the quality, reliability, and robustness of these assessments are critical in order to inform effective decisions and select appropriate solutions for improving environmental performance [66]. In addition, the background systems used through the Ecoinvent and Agribalyse databases are prone to uncertainty since processes are not specific to the conditions of the system under study. Another limitation, not particularly of this study, but generally of LCA, occurs during the design and application of the methodology, when the modeler has alternative approaches from which to choose, leading to widely varying results from case to case, for example, with respect to the environmental impact categories [67]. The methods, as well as indicators involved in this study, were considered appropriate to address a WEF nexus approach, as specified in Section 2.3. However, other methods, such as CED (cumulative energy demand) [68], CML 2001 [69], or WSI (water stress index) [70] are alternatives that would lead to different, but equally valid results.

**Figure 9:**
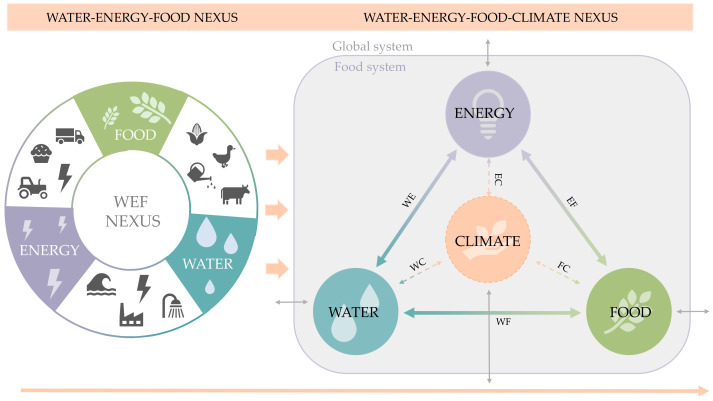
Current nexus approach vs. water–energy–food–climate nexus. Double-arrow lines represent the connection between the pillars and the global environment.

These limitations open the door to future research, particularly in the direction of expanding the WEF nexus approach and LCA application using different environmental impact categories that allow for greater accuracy in the analysis of the performance of food supply chains, which would promote sustainability within a context of globalization of the food sector. Some authors have highlighted a nexus approach based on a combination of water, energy, food, and climate (WEFC) issues. Laso et al. [71] modified the main aspects considered in the nexus to accommodate other issues deemed relevant, such as climate change. On the other hand, Armengot et al. [23] complemented the WEF issue analysis through the evaluation of 10 additional indicators, enabling an understanding of the complex links between these three fundamental pillars and other environmental problems. In this context, future projects could be based on this point, turning the methodology applied in this work into a more complex one, as illustrated in Figure 9, thus obtaining a broader vision not only of this production system, but of any food product.

## 6. Conclusions

This study addressed the water–energy–food nexus issues by analyzing the environmental impacts and nutritional quality of artisanal potato chips. The joint application of LCA and the NRF9.3 index enabled a better understanding of how the product affects consumers’ health and environmental degradation in terms of GHG emissions, eutrophication potentials, and water and energy consumption, helping producers and consumers in the decision-making processes. The outcomes indicated a water use of 3.26 m^3^ per FU, defined as a 50 g bag of potato chips. Eutrophication potentials accounted for 2.85 × 10^−5^ kg P eq./FU and 9.11 × 10^−4^ kg N eq./FU for freshwater and marine water, respectively, whereas energy-related indicators reached 98.2 g CO_2_ eq. for CC and 1.44 MJ per FU for RU. The cultivation and processing stages were identified as the main contributors to environmental burdens; hence, improvement opportunities were focused on these critical points. The application of drip irrigation would lead to significant enhancement of the sustainability of the system, especially in the water footprint, as well as the substitution of diesel by natural gas or pellets, which would reduce the impact in terms of EPm, CC, and RU. In light of these results, the involvement of all stakeholders of the food supply chain is essential to promote the transition and achieve the transformation of the food sector. Farmers have a key role to play in setting an example by applying cleaner agricultural techniques and making efficient use of natural resources. Likewise, processors have the opportunity to implement low-impact technologies and fuels, as well as to avoid waste generation and rely on a circular economy. Transportation has no important impact on this system, which highlights the importance of considering the use of local resources and producing and consuming zero-kilometer products. On the other hand, consumers can do their bit to promote healthy and sustainable consumption habits. The NRF9.3 score of potato chips was calculated as 18.39, which evidences that they are not a completely healthy food, and that their consumption is only recommended occasionally, as is the case in practice, since they are a snack that is not consumed on a regular basis. However, an evaluation scenario of a hot-air fryer showed that, although it causes more environmental degradation than the conventional fryer, the nutritional quality of the final product (roasted potatoes) is significantly better (61.57). This type of information could be decisive in consumers’ decisions. To this end, an ecolabel with a nexus approach that encompasses both the environmental performance and the nutritional quality of a product is advisable, as it would allow consumers to switch to healthier dietary habits, as well as facilitate the process of designing more sustainable consumption patterns. Therefore, raising public awareness and involving the public in decision making along the food supply chain by taking into account their preferences are key steps that represent major challenges for the future.

## Figures and Tables

**Table 1:** LCI for the production of Cantabrian potato chips. All data are provided by FU (50 g bag of potato chips).

Cultivation—Inputs from Technosphere					
Material	Unit	Value	Material	Unit	Value
Potato seeds	kg	1.60 × 10^−2^	Insecticide	kg	2.00 × 10^−6^
Water	L	40.00	Diesel	L	7.20 × 10^−4^
Fertilizer	kg	2.24 × 10^−3^			
**Cultivation—Inputs from Nature**			**Cultivation—Outputs to Technosphere**		
Material	Unit	Value	Product	Unit	Value
Land	Ha	8.00 × 10^−6^	Harvested potatoes	kg	2.00 × 10^−1^
**Transport—Inputs from Technosphere**					
Material	Unit	Value			
Raw potatoes	kg × km	6.00 × 10^−1^			
**Processing—Inputs from Technosphere**					
Material	Unit	Value	Material	Unit	Value
Electricity production	kWh	6.50 × 10^−4^	Salt	kg	1.00 × 10^−4^
Electricity packaging	kWh	3.90 × 10^−3^	Cardboard	kg	1.13 × 10^−3^
Water	L	3.24 × 10^−2^	Polypropylene	kg	1.62 × 10^−3^
Sunflower oil	L	1.66 × 10^−2^	Film	kg	3.20 × 10^−6^
**Processing—Outputs to Technosphere**					
Product	Unit	Value	Product	Unit	Value
Potato chips (50 g bag)	kg	5.00 × 10^−2^	Potato chips (140 g bag)	kg	1.00 × 10^−1^
Used oil	L	1.62 × 10^−3^			
Waste to treatment	Unit	Value	Waste to treatment	Unit	Value
Potato discards	kg	3.15 × 10^−3^	Cardboard	kg	2.40 × 10^−4^
Film	kg	3.20 × 10^−6^	Wastewater	L	3.24 × 10^−2^
**Distribution—Inputs from Technosphere**			**Consumption—Outputs to Technosphere**		
Material	Unit	Value	Waste to treatment	Unit	Value
Potato chips bags	kg·km	5.00	Polypropylene	kg	1.62 × 10^−3^

**Table 2:** Nutrient content of potato chips, DVs, and MRVs for a 2000 kcal daily intake.

	Potato Chips ^a^	DV	MRV ^b^
Protein	6.5 g	50 g ^b^	-
Fiber	4 g	25 g ^c^	-
Vitamin A	0 µg	800 µg ^b^	-
Vitamin C	10 mg	80 mg ^b^	-
Vitamin E	4 mg	12 mg ^b^	-
Ca	37 mg	800 mg ^b^	-
Fe	2 mg	14 mg ^b^	-
K	1190 mg	2000 mg ^b^	-
Mg	50 mg	375 mg ^b^	-
Saturated fats	7.7 g	-	20 g
Added sugar	0 mg	-	50 mg
Na	700 mg	-	2400 mg

^a^ [42]; ^b^ [43]; ^c^ [44].

## Appendix A

**Table A1:** Equivalence between foreground data and Ecoinvent and Agribalyse database processes.

Foreground Data	Process of the Ecoinvent or Agribalyse Databases
Potato seeds	Potato seed, at farm {GLO} productionAPOS, U
Water	Tap water {ES} market forAPOS, U
Fertilizer	Potassium nitrate {RER} market for APOS, U Fertilizing, by broadcaster {GLO} market for APOS, U
Pesticide	Pesticide, unspecified {RER} production APOS, U Application of plant protection product, by field sprayer {GLO} market for APOS, U
Diesel (agricultural machinery)	Diesel, burned in agricultural machinery {GLO} diesel, burned in agricultural machinery APOS, U
Land	Transformation, from permanent crop, irrigated, extensive
Tractor (transport)	Transport, tractor and trailer, agricultural {CH} market for transport, tractor and trailer, agricultural APOS, U
Electricity	Electricity, medium voltage {ES} market for APOS, S
Sunflower oil	Sunflower oil, processed in FR Ambient (long) PET at supermarket/FR
Salt	Salt {GLO} market for salt APOS, U
Diesel (generator)	Diesel, burned in diesel-electric generating set, 18.5 kW {GLO} diesel, burned in diesel-electric generating set, 18.5 kW APOS, U
Cardboard	Corrugated board box {RER} market for corrugated board box APOS, U
Polypropylene	Polypropylene, granulate {RER} production APOS, U
Film	Packaging film, low density polyethylene {RER} production APOS, U
Van (distribution)	Transport, freight, lorry 3.5–7.5 metric ton, euro6 {RER} market for transport, freight, lorry 3.5–7.5 metric ton, EURO6 APOS, U
Wastewater treatment	Wastewater, average {Europe without Switzerland} market for wastewater, average APOS, U
Cardboard waste treatment	Waste paperboard {ES} market for waste paperboard APOS, U
Polypropylene waste treatment	Waste polypropylene {ES} market for waste polypropylene APOS, U
